# Advanced Practice Pharmacists: a retrospective evaluation of the efficacy and cost of ClinicaL Pharmacist PractitionErs managing ambulatory Medicare patients in North Carolina (APPLE-NC)

**DOI:** 10.1186/s12913-016-1851-2

**Published:** 2016-10-21

**Authors:** Michèle M. Kislan, Adam T. Bernstein, Loretta R. Fearrington, Timothy J. Ives

**Affiliations:** 1 Yakima Valley Memorial Hospital, Yakima, WA 98902 USA; 2 Department of Pharmacy, University of North Carolina Health Care, Chapel Hill, NC 27514, USA; 3 The North Carolina Translational & Clinical Sciences Institute, University of North Carolina at Chapel Hill, Chapel Hill, NC 27599-7064 USA; 4 Eshelman School of Pharmacy, University of North Carolina at Chapel Hill, Chapel Hill, NC 27599-7574 USA; 5 Division of General Medicine and Clinical Epidemiology, School of Medicine, University of North Carolina at Chapel Hill, Chapel Hill, NC 27599-7110 USA

**Keywords:** Ambulatory care, Pharmacy, Clinical pharmacist practitioner, Medicare

## Abstract

**Background:**

Clinical Pharmacist Practitioners are advanced practicing pharmacists in North Carolina that provide disease-specific management. The purpose of this retrospective cohort study was to compare the efficacy and charges from referrals to a Clinical Pharmacist Practitioner by the primary care provider, to those managed by a primary care provider alone.

**Methods:**

Patients were separated into cohorts depending if they had at least two appointments with a Clinical Pharmacist Practitioner from November 2008 to November 2011. A primary care provider saw all patients at least twice during the study period. Cohorts were then matched by age, gender, and disease states. Medicare billed data was evaluated from outpatient visits related to hypertension, diabetes mellitus, and peripheral neuropathy, as well as emergency department visits and inpatient admissions. Cost of medications was estimated using 2009 AWP data corresponding to medication histories within the electronic medical record. Efficacy was defined as ability to reach disease state goal determined using national guidelines and reduction in pain score. Efficacy was analyzed by difference-in-differences test and all other numerical data tested by paired *t*-tests.

**Results:**

The Clinical Pharmacist Practitioners cohort experienced more outpatient visits (1338 vs. 858, *p* < 0.001), fewer emergency department visits (115 vs. 190, *p* < 0.05), and similar inpatient admissions (88 vs. 117, *p* > 0.05) than the primary care providers cohort, respectively. The Clinical Pharmacist Practitioners cohort showed changes in charges of +22.6 % for outpatient visits, −45.5 % emergency department visits, and −13.2 % inpatient admissions relative to the primary care provider cohort. There was no difference in average daily medication cost (Clinical Pharmacist Practitioners $38.52 vs. primary care providers $38.23, *p* = 0.97) or achievement of disease state goals.

**Conclusion:**

APPLE-NC demonstrated that through referrals, Clinical Pharmacist Practitioners provide services comparable in charges and efficacy to primary care providers. Consequently, the current increased need for primary care practitioners can be met in part by increasing the utilization of advanced practice pharmacists for chronic disease management.

**Trial registration:**

This does not apply for this retrospective cohort study.

**Electronic supplementary material:**

The online version of this article (doi:10.1186/s12913-016-1851-2) contains supplementary material, which is available to authorized users.

## Background

The implementation of the Affordable Care Act has increased the eligibility of many Americans to receive healthcare benefits resulting in a shortage of primary care physicians [1]. Increasing use of non-physician providers, such as family nurse practitioners (FNPs) and physician assistants (PAs), to provide primary care services to patients may reduce the need for additional primary care physicians [1]. Advanced practice pharmacists, who by individual state regulations, can manage referred patients by initiating, changing, and discontinuing mediations for their patients, including controlled substances.

Collaborative Drug Therapy Management (CDTM), whereby a physician and a pharmacist establish a collaborative practice agreement in which the pharmacist can initiate, modify or continue drug therapy for a patient in that practice, is permitted in 48 states and the District of Columbia [2]. However, only four states allow pharmacists with prescriptive powers to manage the pharmacotherapy of referred patients: California, Montana, New Mexico, and North Carolina [3, 4]. These states require supplemental education and certification, including diagnosis and physical assessment training, for advanced practice pharmacists [5].

In North Carolina, advanced practice pharmacists are called Clinical Pharmacist Practitioners (CPPs). The North Carolina Medical Practice Act, which states that any pharmacist who is approved to perform medical acts, tasks, and functions may use the title *Clinical Pharmacist Practitioner*, was enacted by the North Carolina General Assembly in 1999 [6, 7]. A pharmacist is required to complete advanced training and to be approved by both the North Carolina Board of Pharmacy and North Carolina Medical Board to qualify for CPP licensure [6, 8]. Currently, ambulatory patients can be referred to a CPP for chronic disease management, thus decreasing their primary care provider (PCP) visits and allowing the PCP to provide medical care to more patients.

Interventions by clinically oriented pharmacists, who have similar responsibilities to CPPs but do not have additional diagnostic, physical assessment and pharmacotherapy management training, have been shown to improve outcomes in patients with chronic diseases, such as Diabetes mellitus (DM), hypertension (HTN), and pain [9–11]. A study of 1016 hospitals in the United States demonstrated a reduction in total costs of care when clinical pharmacy services were present [12]. In a recently published systematic review, clinical pharmacy services were shown to be cost-effective or to provide a good benefit-to-cost ratio [13].

The purpose of the APPLE-NC study was to establish the clinical efficacy and charges from CPP referred interventions, as compared to interventions provided by primary care providers (PCP: physicians, FNPs, and PAs) through evaluation of outpatient visits (OPV), emergency department visits (EDV), inpatient admissions (IA), and medications prescribed in chronic disease management of Medicare recipients. This study demonstrates the utilization and value associated with CPP interventions in a sample of Medicare patients by analyzing patient chronic disease management and the charges associated with that care.

## Methods

### Design

This was a retrospective, matched cohort study, from November 2008 to November 2011. Participants were Medicare beneficiaries enrolled at University of North Carolina (UNC) Health Care, and seen at one of the UNC multidisciplinary outpatient clinics. Patients were seen by either the CPP as a referral from the patient’s PCP (CPP-R), or the PCP alone (PCP-A). Physician groups at UNC Healthcare System clinics holding formal relationships with CPPs were utilized in this analysis.

Parameters collected to determine the impact of CPP-provided patient care on overall expenditures included clinical outcomes, laboratory results, number of OPV, number of EDV and IA, medications and charges associated with each visit and admission. Medicare billed data for OPV, EDV, and IA were provided by the UNC Clinical Data Warehouse for Health (CDW-H) [14].

### Inclusion and exclusion criteria

All Medicare patients who were internally referred to the UNC outpatient clinics utilizing CPP services were eligible for inclusion. All patients required an ICD-9 code for HTN, DM, or peripheral neuropathy (PN) in their claims data. These disease states were selected because of their clinical interrelatedness, prevalence in the United States, and the associated complexity of their pharmacotherapy management. Patients were included in the CPP-R cohort if they saw a CPP at least twice during the 3-year study period, in addition to at least two PCP visits. Patients were included in the PCP-A cohort if they were not managed by a CPP and required at least two visits to a PCP during the study period. Dual Medicare/Medicaid recipients were excluded from this study because costs paid by Medicaid could serve to confound the costs associated with care and would render the study less generalizable to other states and health care systems.

### Study outcomes

The primary study outcomes were the percentage of patients who reached their disease state goal, such as blood pressure (BP) <140/90 mmHg for patients with HTN, or glycosylated hemoglobin (HgbA1c) <7.0 % for patients with DM, or achieving a patient’s desired pain goal. These goals were determined by the patient’s PCP based on the patient’s age and comorbidities by using national or international guidelines for management of HTN and DM, such as the Seventh Report of the Joint National Committee on Prevention, Detection, Evaluation, and Treatment of High Blood Pressure (JNC-7), the International Society of Hypertension Guidelines, or the American Diabetes Association Standards of Medical Care in Diabetes.

The secondary outcomes were the number of and percent change of charges of OPV, IA, and EDV for any diagnosis, estimated cost of medications prescribed, and the average number of therapy changes per year defined as a dose increase or decrease, or drug initiation or discontinuation. EDV and IA related to management of HTN, DM, and pain were included in this study.

Clinical efficacy was determined by how well-controlled patients’ disease states were with respect to biomarkers and indicators, as compared with disease state goals set at the baseline study visit. Baseline measurements of clinical biomarkers (HgbA1c, BP) and indicators (Brief Pain Inventory score (BPI) were determined at the first visit within the start of the 36-month period, provided that the first visit was within six months from the beginning of the study period. If the visits began later than May 1, 2009, the baseline systolic BP was recorded from a routine visit within 6 months of the beginning of the study period at a clinic, or appointment that was not in the previously specified clinics. These biomarkers were also recorded at the last visit within the study period as an endpoint for determining clinical efficacy.

The BP, HgbA1c, and BPI values were not recorded for baseline characteristics or for the last visit if the patient was seen at the visit for an infectious illness, due to its acute impact on these values. Urgent care, ED, and inpatient biomarkers and indicators were also excluded from baseline characteristics and from last visit data collection as these visits may have reported elevated BP, HgbA1c, and BPI scores due to an emergent, severe, or febrile illness, which were not representative of chronic disease management. If the BP was noted to be unusually low or high, and the PCP recorded a second reading, both BP readings were averaged for a final BP reading that was included in the study. For patients that missed their morning BP medications, the BP from the next available appointment was used.

Each patient was assessed individually for the BP goal set at the previous appointment to determine if the current provider was able to meet that goal. When a goal was not available in the medical record, the Seventh Report of the Joint National Committee on Prevention, Detection, Evaluation, and Treatment of High Blood Pressure (JNC-7) was consulted for BP values for patients with HTN only, and the American Diabetes Association Standards of Medical Care in Diabetes (ADA guidelines) 2008 through 2011 were consulted for appropriate HgbA1c and BP goals [15–19]. ADA guidelines from the appropriate year were followed for patients with concurrent DM and HTN.

Pain is subjective, and management goals are patient-specific. A decrease in pain score or level is a general goal of treatment, however, the specific decrease in score or level differs between patients. Pain intensity scores were assessed at the first and last visits, and determined to be at goal if the pain score decreased by at least one point on a scale of 1–10 [20]. A decrease in at least two points in the pain intensity score was also analyzed.

Clinical efficacy was achieved if the biomarkers and indicators improved or decreased at the last visit within the study period, compared to the initial or baseline measurement (e.g., a pain score of nine at the first visit in the study, to a pain score of eight at the last visit in the study). Measurements that remained unchanged at the beginning and at the end of the study period were not considered an improvement.

The number of disease-related EDV and IA were determined by screening the medical records of each patient. HTN- and DM-related events and sequelae are listed in the Additional file 1. Patients who did not have HTN, DM, or PN were excluded from this analysis.

Patients were included in the charge analysis if they received any program interventions through the UNC outpatient clinics. Direct medical charges were defined as the amount charged to Medicare by the patient’s health plan for routine office visits, EDV, IA, procedures, and other ancillary services. Charges associated with the patient’s covered visits were current for the date of the visits. Therefore, charges were not annualized.

Medicare charges were also obtained for OPV, EDV, and IA. As all costs were reported to Medicare at the time of service, no adjustments were made for inflation. The medical costs associated with the HTN-, DM-, and chronic pain-related EDVs and IAs were determined from CDW-H claims data.

### Medication history

Prescribed medications were tracked using WebCIS, an electronic medical record (EMR) utilized by UNC Health Care during the study period. Only outpatient prescription medications were included for cost analysis. Herbal supplements, vitamins, minerals and medications only available over-the-counter were excluded.

### Medication and total cost calculation

All medications were priced according to 2009 average wholesale price (AWP) listed within the 2010 edition of Redbook [21]. The manufacturer with the lowest AWP was used for each medication. Manufacturers that repackage medications list a wide range of AWPs. Thus, repackagers were excluded to reduce variability when comparing drug costs. All medications were assumed to be generic unless only brand was available during 2009. Medication cost was calculated by multiplying the number of drug units used during the treatment duration by the AWP. Drug unit was defined as a tablet, milliliter of solution, box of medication (e.g., inhaler, topical products). Medications prescribed on an as needed basis were priced as a one-month supply if the prescription directions recorded in the EMR indicated exact quantities. If these PRN medications did not indicate quantities, they were excluded from pricing and were only evaluated for therapy changes. Treatment duration was defined as time from first to last recorded medical visit. Medication adherence was assumed to be 100 % for analysis of the data. Adherence rates are likely different for patients receiving comprehensive services from pharmacists compared to those only seen by primary care providers, however these rates have not been well established in the literature. Therefore, an adherence rate of 100 % was used to ensure consistent analysis of data in both cohorts without introducing additional bias. The total cost was calculated by adding total estimated medication costs with OPV, EDV, and IA charges for any chief complaint. This total cost was divided by the number of days seen by a provider to generate cost per day. Cost was standardized with time to prevent the appearance of more expensive treatment due to longer duration of care within the study period. Similarly, total therapy changes were standardized by year.

### Analysis

Matched cohorts were created to reduce confounding variables. ICD-9 codes for HTN, DM, and PN were used to create a pool of patients eligible for randomization. The 250.XX code was used to include patients with diabetic complications, which resulted in inclusion of Type 1 DM and Type 2 DM in this study.

Subjects were matched by age, gender, and disease state using SAS software, version 9.2 (SAS Institute, Inc., Cary, NC (2008)). One hundred matches were randomly generated and selected for inclusion into the study, using a random number generator in Microsoft Excel 2013 (Microsoft, Redmond, WA). A power calculation for the primary outcome using Power and Precision, version 2 (Biostat, Engelwood, NJ) found that a sample of 65 patients in each cohort would be needed to detect an effect of 0.5, as defined by Cohen, with 80 % power using a two-tailed *t*-test and an alpha of 0.05 [22]. The first 65 patient pairs were chosen to meet minimum power. Efficacy endpoints were analyzed using a difference-in-differences test and all other numerical data, such as difference in average medication cost per day per patient, were analyzed using a paired *t*-test.

Results from the analysis of charges are reported as a percent difference due to proprietary rights of charges billed to patients by the UNC HealthCare System. This study was approved by the Institutional Review Board of the Office of Human Research Ethics at the University of North Carolina at Chapel Hill.

## Results

### Demographics

During the study period, 12,957 patients were available for inclusion into this study, and 503 patients were seen by a CPP at least twice. When matched to the 12,065 patients in the PCP-A group, 378 patients were matched according to gender, age, and disease state. Of these, 65 pairs were selected randomly and included for analysis to meet minimum power while ensuring feasibility of study completion. Although the data was matched using ICD-9 codes, there were several discrepancies in the medical records stating that a patient did or did not have HTN, DM, or PN.

Patient characteristics were similar between both cohorts (Additional file 1: Table S1). Baseline characteristics of the CPP-R and PCP-A cohorts resulted in no statistically significant differences regarding BMI (*p* = 0.85), average 10-year Atherosclerotic Cardiovascular Disease (ASCVD) risk (*p* = 0.80), total serum cholesterol (*p* = 0.86), and serum high density lipoprotein (*p* = 0.55) (Table 1). Measurements of SBP and BPI recorded from the first study visit were also non-significant between the two cohorts (*p* = 0.27) (Table 1), however, significantly higher baseline HgbA1c levels were recorded in the CPP-R cohort compared to the PCP-A cohort (7.5 vs. 6.8, *p* = 0.02) (Table 1).

**Table 1:** Clinical characteristics of patients at baseline

	CPP (n = 65)		PCP (n = 65)		
	Mean	95 % CI	Mean	95 % CI	*p*-value
BMI	31.2	29.4–33.0	31.0	29.3–32.6	0.85
Average 10 year CVD risk	23.3	18.1–28.5	22.7	17.8–27.7	0.80
Systolic Blood Pressure Average	132.4	127.3–137.5	136.6	131.2–142.0	0.27
Total Cholesterol	184.2	169.4–199.0	182.4	168.0–196.8	0.86
High Density Lipoprotein	51.2	46.6–55.8	53.3	48.2–58.3	0.55
Hemoglobin A1c	7.5	6.8–8.2	6.8	6.2–7.3	0.02
Brief Pain Inventory Score	7.2	6.2–8.2	6.5	5.0–8.0	0.37
Outpatient Visits^a^	20.6	18.2–22.9	13.2	10.5–15.9	0.0002
Primary Care Provider Visits	15.0	12.9–17.1	13.2	10.5–15.9	0.32
Clinical Pharmacist Practitioner Visits^b^	5.6	4.6–6.7	0	N/A	N/A
Emergency Department Visits^c^	1.7	1.2–2.3	2.9	1.7–4.1	0.06
Emergency Department Visits related to HTN, DM, Pain^d^	0.25	0.1–0.4	0.52	0.10–0.95	0.20
Inpatient Admissions^e^	1.4	0.8–1.9	1.8	1.0–2.6	0.32
Inpatient Admissions related to HTN, DM, Pain^f^	0.5	0.1–0.9	0.4	0.2–0.5	0.51

^a^Total outpatient visits: CPP cohort 1338 visits; PCP cohort 858 visits

^b^Patients in the PCP cohort did not receive treatment from CPPs, therefore, there were no CPP visits in the PCP, and a *p*-value was unable to be calculated

^c^Emergency Department visits: CPP 115 ED visits; PCP 190 ED visits

^d^Emergency Department visits related to HTN, DM, Pain: CPP 16 ED visits; PCP 34 ED visits

^e^Inpatient Admissions: CPP 88 IA; PCP 117 IA

^f^Inpatient Admissions related to HTN, DM, Pain: CPP 32 IA; PCP 23 IA

### Visits and charges

Average OPV per patient in the CPP-R cohort were significantly higher than the total number of OPV per patient in the PCP-A cohort (20.6 vs 13.2, respectively; *p* = 0.0002) (Table 1). The average charge associated with an outpatient CPP-R visit was 20.7 % lower than the average charge associated with an outpatient PCP-A visit (Table 2).

**Table 2:** Percent change of charges associated with outpatient visits, emergency department visits, and inpatient admissions between the CPP and PCP cohorts

	% Change
Outpatient Visits	
Outpatient Visit Charges	22.6
Charges per Visit	−20.7
Emergency Department Visits	
Total Emergency Department Visits Charges	−45.6
Emergency Department charges related to HTN, DM, Pain	21.0
Inpatient Admissions	
Total Inpatient Charges	−13.2
Inpatient Charges related to HTN, DM, Pain	30.4

Due to proprietary concerns regarding Medicare charges, percent change was used to show the difference in charges between the CPP and PCP cohorts. If the value in the table above is negative, then the CPP cohort variable is n% less than the PCP cohort variable. The converse is true for a positive number: the CPP cohort variable is n% more than the PCP cohort. For example, the CPP cohort resulted in 22.6 % higher outpatient visit charges and 20.7 % lower outpatient charges per visit than the PCP cohort

$$ \mathrm{Percent}\ \mathrm{C}\mathrm{hange} = \frac{\left(\mathrm{C}\mathrm{P}\mathrm{P}\ \mathrm{value}\ \hbox{--}\ \mathrm{P}\mathrm{C}\mathrm{P}\ \mathrm{value}\right)}{\left(\mathrm{C}\mathrm{P}\mathrm{P}\ \mathrm{value}\right)}\times 100 $$

Patients who received health care services from a CPP visited the ED less frequently, but this difference was not statistically significant (1.7 vs 2.9, respectively; *p* = 0.06) (Table 1). The total charge for EDV for any diagnosis, however, was 45.6 % lower in the CPP-R group (Table 2).

Patients in the CPP-R cohort were admitted to an inpatient service with an average of 1.4 IA vs. 1.8 IAs in the PCP-A cohort, but the difference was not statistically significant (*p* = 0.32) (Table 1). Total IA charges associated with CPPs was 13.2 % lower than charges in the PCP-A cohort, however charges associated with IA related to HTN, DM, and/or pain in the CPP-R cohort resulted in costs that were 30.4 % higher than in the PCP-A cohort (Table 2).

### Clinical efficacy

More patients were at or below their BP goal in the CPP-R cohort at the beginning and at the end of the study period than the PCP-A cohort, however the difference between the two cohorts did not achieve statistical significance (*p* = 0.48) (Table 3). At the end of the study period, patients treated by CPPs reached their HgbA1c goal as often as patients in the PCP-A cohort (*p* = 0.21). Pain scores decreased by one point on the pain scale in 26 patients (55.3 %) in the CPP-R cohort, compared to 13 patients (41.9 %) in the PCP-A cohort, which was not statistically significant (*p* = 0.30). A decrease by two points on the pain scale was also not significantly different between cohorts (*p* = 0.47) (Table 3).

**Table 3:** Clinical efficacy of CPPs compared to PCPs

	CPP	PCP	*p*-value*
Number (%) of patients with BP at goal			
Beginning of study	36 (57.1)	30 (47.6)	0.48
End of Study	40 (63.5)	29 (46.0)	
Number (%) of patients with HgbA1c at goal			
Beginning of study	2 (7.1)	22 (61.1)	0.21
End of Study	7 (25.0)	23 (63.9)	
Number (%) of patients with pain at goal			
BPI score decreased by 1 point	26 (55.3)	13 (41.9)	0.30
BPI score decreased by 2 points	20 (40.4)	10 (32.2)	0.47

*p-value calculated using Difference in Differences test

### Medication cost per cohort

No statistical difference existed in the mean difference of medication cost per day per patient between both cohorts. The average medication cost per day per patient was $38.52 in the CPP-R cohort, and $38.23 per day per patient for the PCP-A patients (*p* = 0.97). Patients in the CPP-R cohort experienced more changes to drug therapies per year on average than patients in the PCP-A patients (21.1 vs. 15.1, respectively; *p* = 0.032) (Table 4).

**Table 4:** Average medication cost per day and therapy changes per year

	CPP	PCP	Mean difference	*P*-value
Average medication cost per day per patient	$ 38.52	$ 38.23	$ 0.29	0.97
Average therapy change per year per patient	21.1	15.5	5.6	0.032

### Total cost

The total cost of OPV, EDV, IA, and prescribed medications in the CPP-R cohort was 1.46 % lower, as compared to the PCP-A cohort (*p* = 0.026) (Data not shown due to proprietary nature of data).

## Discussion

Patient characteristics were similar between both cohorts, however significantly higher baseline HgbA1c levels were recorded in the CPP-R cohort as compared to the PCP-A cohort. Lower charges were accrued by the CPP-R cohort for their OPV, but visited the outpatient clinics significantly more than patients in the PCP-A cohort. There was no statistical difference between the EDV and IA between cohorts, and overall charges accrued for EDV and IA were lower in the CPP-R cohort. Patients reached their specific disease state goal in both cohorts with no difference in the medication costs per day for patients. The CPP-R cohort required more drug therapy changes than the patients in the PCP-A cohort. The overall cost of OPV, EDV, IA, and prescribed medications in the CPP-R cohort was significantly lower than the overall cost in the PCP-A cohort.

Pharmacists are the most accessible healthcare profession and are considered to be underutilized to the extent of their education [23]. Pharmacists are a cost-effective addition to the health care team, as their pharmacotherapeutic knowledge improves patient outcomes and decreases adverse events [5]. To date, most research has focused on the benefits of clinical pharmacy services, such as the Asheville Project in North Carolina [24]. More recently, research on a pharmacist-PCP collaborative effort in California demonstrated better health outcomes for their patients than when utilizing only a PCP [25].

In practice, CPPs often manage high-risk patients with multiple comorbidities, complex drug regimens, or patients refractory to standard therapy [26, 27]. Patients referred to clinical pharmacists by PCPs generally have multiple comorbidities, or are refractory to standard of care medications, as provided by their PCP [28–30]. As one example, the HgbA1c percentage of the patients in the CPP-R cohort was significantly higher (*p* = 0.02) than patients in the PCP-A cohort at the outset of the study. Patients in the CPP-R cohort demonstrated more frequent OPV, EDV, and IA than the patients in the PCP-A cohort, which may be indicative of more complicated illnesses, or refractory-to-standard treatment disease states in patients that were referred to a CPP.

The difference in OPV charges is most likely due to lack of recognition of pharmacists or CPPs as health care providers under Medicare, and therefore, the inability for CPPs to bill at Current Procedural Terminology (CPT) codes higher than 99211 CPT code. The higher cost associated with EDV and IA in the CPP-R cohort is most likely related to more complex disease states requiring more expensive treatment, or treatment for a longer period of time.

In this study, clinical interventions by CPPs resulted in patients reaching their BP, HgbA1c, and pain goals as often as the PCP-A patients. Although BP and HgbA1c goals were easy to measure, pain is subjective and difficult to treat due to its complexity of genetic, environmental, social, and cognitive variables [31]. Due to the complexity of pain management and lack of individualized pain goals in the medical chart, clinical efficacy was determined if the pain scores decreased by at least one point at the end of the study period. A one to two point reduction is considered the minimally accepted threshold for determining clinical importance of chronic pain management outcomes, based upon the IMMPACT researchers’ findings in 2008 [20].

A recent retrospective pre-post study analysis revealed clinical and cost-benefits of added CPPs in a neurotrauma intensive care unit setting [27]. The additional CPPs provided therapeutic interventions and prevented adverse drug events, resulting in an estimated 30 % in cost-savings [27]. In this study, the overall charges for health care were demonstrated to be higher in the PCP-A cohort, although the charges associated with the management of HTN, DM, and/or chronic pain in the CPP-R cohort was higher. This suggests that patients in the CPP-R group were more complex and had more complicated medical conditions that necessitated higher medical charges during EDVs and IAs.

The medication arm of the APPLE-NC study provided an estimate of the total cost of medications prescribed to PCP-A study patients, compared to those also managed by CPPs. There was no significant difference in medication cost between the two cohorts despite more pharmacotherapy changes in the CPP-R cohort. The non-significant difference between the cohorts may be due to utilization of additional, lower cost medications to optimize pharmacotherapy, more dose adjustments to optimize therapy, or pharmacotherapy adjustments that may not contribute to overall cost of medications.

With the inception of the Affordable Care Act, there is an emphasis on finding affordable and effective health care. Implementation of pharmacist-driven services represent one possible solution to this problem, however, lack of reimbursement limits the utility of pharmacists as advanced practicing providers. Currently, there is momentum in Congress for recognition of pharmacists as providers, with legislation such as the Pharmacy and Medically Underserved Areas Enhancement Act (H.R. 592), which would amend title XVIII of the Social Security Act to cover pharmacist services. Additional cost and outcomes data will aid the passing of federal legislation that enable pharmacist reimbursement for the clinical services they currently provide, however, limited data exists to compare the cost-effectiveness of pharmacist interventions to those of PCPs. This study contributes to the efforts for provider status by demonstrating advanced practicing pharmacists can provide chronic hypertension, diabetes mellitus, and chronic pain management in ambulatory care patients that matches those of PCPs.

### Limitations

This study has several limitations to the interpretation of the data. First, a small sample size was specifically utilized in this retrospective cohort study. Also, medical visits at facilities unaffiliated with UNC Health Care were not used in this data analysis. Some data, such as HTN or DM goals, were not reported in medical notes because of omission or they were determined prior to the study period. For the purposes of this study, goals were determined by assessing each patient’s health status, comorbidities, and the setting of a goal according to the guidelines set forth by JNC-7 and ADA guidelines when not available in medical notes.

Selection bias may have existed because patients referred to CPPs may have been more refractory to treatment that was provided by the PCP alone, which is considered as a standard of care. This bias may have skewed study results due to variations in patient complexity, such as seen with the higher HgbA1c levels. Also, medication adherence issues cannot be excluded with either cohort. It is assumed that patients prescribed medications were adherent to prevent introducing bias into the analysis, however, it is outside the scope of this study to ascertain medication adherence in this retrospective analysis.

Although data were matched using ICD-9 codes, the electronic health records documentation did not show a HTN, DM, or PN diagnoses for several patients in the cohort. This resulted in an unequal matching of disease states between the cohorts, however the number of patients in each cohort remained the same and did not affect the primary outcome. Documentation of the term “peripheral neuropathy” or “PN” in the electronic health record was poor or absent in patients with the ICD-9 codes for PN, and often PN was described as “pain” or “chronic pain” in the electronic health record. Therefore, BPI scores for chronic pain, documented in patients with ICD-9 codes for HTN, DM, and PN, were included in analysis. All patients were assessed for their level of pain on a 0–10 scale at the beginning of their OPV, and all patients were included in the study, provided that the pain was chronic and managed by their CPP or PCP. Pharmacies and healthcare systems may obtain medications at prices lower than AWP, depending upon contractual agreements. The AWP was limited to one year, 2009, to reduce the effect of AWP variability that may not translate directly to medication price fluctuations to the consumer or third party plan.

## Conclusion

In Medicare-eligible patients in North Carolina included in the APPLE-NC study, CPPs were shown to be as effective as PCPs in chronic disease management. Study patients tended to have higher HgbA1c levels, and were more refractory to treatment, as demonstrated by the higher emergency department and inpatient costs associated with HTN, DM, and chronic pain. This study demonstrated that CPPs decreased overall EDV and IA, and enabled their patients to reach BP and HgbA1c goals, as well as to manage pain and to decrease pain scores. Consequently, the current increased need for PCPs can be met in part by increasing the utilization of advanced practice pharmacists for chronic disease management thereby allowing PCPs to provide care for new patients or highly complex disease states. It is anticipated that this study will motivate other health care systems across the United States to replicate these results, provide justification for expanding the scope of pharmacy practice, and continue to improve the quality and value in the provision of patient care.

## Additional file

Additional file 1:
**Table S1.** Patient Demographics at Baseline. **Table S2.** List of Emergency Department Visit Diagnoses related to Hypertension, Diabetes Mellitus, and/or Chronic Pain^. **Table S3.** List of Inpatient Admission Diagnoses related to Hypertension, Diabetes Mellitus, and/or Chronic Pain^. (DOCX 35.7 kb)

## Abbreviations

ADA: American Diabetes Association
ASCVD: Atherosclerotic cardiovascular disease
AWP: Average wholesale price
BA: Bachelor of Arts
BP: Blood pressure
BPI: Brief pain inventory
CDTM: Collaborative Drug Therapy Management
CPP: Clinical pharmacist practitioner
CPT: Current procedural terminology
DM: Diabetes mellitus
EDV: Emergency department visit
EMR: Electronic medical record
FNP: Family nurse practitioner
HgbA1c: Hemoglobin A1c
HTN: Hypertension
IA: Inpatient admission
JNC-7: Joint National Committee
MPH: Master of Public Health
OPV: Outpatient visit
PA: Physician assistant
PCP: Primary care provider
PharmD: Doctor of Pharmacy
PN: Peripheral neuropathy
UNC: University of North Carolina
