# Erratum to: Advanced Practice Pharmacists: a retrospective evaluation of the efficacy and cost of ClinicaL Pharmacist PractitionErs managing ambulatory Medicare patients in North Carolina (APPLE-NC)

**DOI:** 10.1186/s12913-016-1923-3

**Published:** 2016-11-28

**Authors:** Michèle M. Kislan, Adam T. Bernstein, Loretta R. Fearrington, Timothy J. Ives

**Affiliations:** 1 Yakima Valley Memorial Hospital, Yakima, WA 98902 USA; 2 Department of Pharmacy, University of North Carolina Health Care, Chapel Hill, NC 27514 USA; 3 The North Carolina Translational & Clinical Sciences Institute, University of North Carolina at Chapel Hill, Chapel Hill, NC 27599-7064 USA; 4 Eshelman School of Pharmacy, University of North Carolina at Chapel Hill, Chapel Hill, NC 27599-7574 USA; 5 Division of General Medicine and Clinical Epidemiology, School of Medicine, University of North Carolina at Chapel Hill, Chapel Hill, NC 27599-7110 USA

## Erratum

Following the publication of this article [1] it was brought to our attention that there was an error in the ‘Total cost’ paragraph of the ‘Results’ section.

The paragraph currently reads: “The total cost of OPV, EDV, IA, and prescribed medications in the CPP-R cohort was 1.46% lower, as compared to the PCP-A cohort (*p* = 0.026) (Data not shown due to proprietary nature of data).”

This should instead read: “The total cost of OPV, EDV, IA, and prescribed medications in the CPP-R cohort was 119.6% lower, as compared to the PCP-A cohort (*p* = 0.026) (Data not shown due to proprietary nature of data).”
