# Supplementary material for: Advanced Practice Pharmacists: a retrospective evaluation of the efficacy and cost of ClinicaL Pharmacist PractitionErs managing ambulatory Medicare patients in North Carolina (APPLE-NC)
Source: BMC Health Serv Res. 2016 Oct 21;16:607. doi: 10.1186/s12913-016-1851-2 (PMC5073726; doi:10.1186/s12913-016-1851-2)
Supplement: Additional file 1: — Table S1. Patient Demographics at Baseline. Table S2. List of Emergency Department Visit Diagnoses related to Hypertension, Diabetes Mellitus, and/or Chronic Pain^. Table S3. List of Inpatient Admission Diagnoses related to Hypertension, Diabetes Mellitus, and/or Chronic Pain^. (DOCX 35.7 kb) [file 12913_2016_1851_MOESM1_ESM.docx]

**Supplementary Data**

**Table S1. Patient Demographics at Baseline**

|  | **CPP (n=65)** | **PCP (n=65)** |
| --- | --- | --- |
| **Female, n (%)** | 40 (61.5) | 40 (61.5) |
| **Male, n (%)** | 25 (38.5) | 25 (38.5) |
| **Age, Average, n** | 64 | 64 |
| **Age < 65, n (%)** | 27 (41.5) | 27 (41.5) |
| **Age ≥ 65, n (%)** | 38 (58.5) | 38 (58.5) |
| **Caucasian, n (%)** | 41 (63.1) | 38 (58.5) |
| **African American, n (%)** | 22 (33.8) | 26 (40.0) |
| **Hispanic, n (%)** | 1 (1.5) | 1 (1.5) |
| **Asian, n (%)** | 1 (1.5) | 0 (0.0) |
| **Hypertension, n (%)** | 63 (96.9) | 63 (96.9) |
| **Diabetes, Type 2, n (%)** | 28 (43.1) | 36 (55.4) |
| **Peripheral Neuropathy, n (%)** | 34 (52.3) | 31 (47.7) |
| **Patients’ disease states, n (%)** |  |  |
| **Hypertension only, n (%)** | 16 (24.6) | 14 (21.5) |
| **Diabetes only, n (%)** | 0 (0.0) | 0 (0.0) |
| **Peripheral Neuropathy only, n (%)** | 2 (3.1) | 2 (3.1) |
| **Hypertension and Diabetes, n (%)** | 15 (23.1) | 20 (30.8) |
| **Hypertension and Peripheral Neuropathy, n (%)** | 19 (29.2) | 13 (20.0) |
| **Diabetes and Peripheral Neuropathy, n (%)** | 0 (0.0) | 0 (0.0) |
| **Hypertension, Diabetes, and Peripheral Neuropathy, n (%)** | 13 (20.0) | 16 (24.6) |
| **Smoker, n (%)** | 11 (16.9) | 11 (16.9) |
| **Non-smoker, n (%)** | 54 (83.1) | 54 (83.1) |
| **Treatment for HTN, n (%)** | 59 (90.8) | 57 (87.7) |

Data are provided as number (percentage). Demographics were used to match patients, inclusion in study was random, and therefore p-values were not calculated.

**Table S2. List of Emergency Department Visit Diagnoses related to Hypertension, Diabetes Mellitus, and/or Chronic Pain^**

| **CPP** | **PCP** |
| --- | --- |
| - Abdominal pain, chronic non-cancer - Back pain, chronic - Chest pain^#^ - Diabetes complications (cellulitis) - Foot pain, chronic - Joint pain, caused by drug induced lupus flare - Headache - Heart failure exacerbation (shortness of breath) - Hypertension - Hypoglycemia - Hypotension | - Back pain, chronic - Cardiomegaly with pulmonary embolism - Chest pain^#^ - Diabetes complications (foot infection, cellulitis, neuropathy) - Headache - Heart Failure exacerbation (edema) - Hypertension - Hypoglycemia - Hypotension - Lumbar pain, chronic - Neck pain, chronic |

^Excluded falls, accidents, lacerations or pain due to cancer or tumors

^#^Not related to myocardial infarction

**Table S3. List of Inpatient Admission Diagnoses related to Hypertension, Diabetes Mellitus, and/or Chronic Pain^**

| **CPP** | **PCP** |
| --- | --- |
| Atrial fibrillation with RVR  Chronic pancreatitis  Diabetic complications (cellulitis, ulcer)  Diabetic gastroparesis  Heart failure  Hyperglycemia  Hypertensive urgency  Hypertension  Hypotension  Intractable pain  Osteoarthritis | Atrial fibrillation with cardioversion  Atrial fibrillation with dofetilide (Tikosyn) load  Back pain  Bradycardia  Chest pain^#^  Diabetic complications (cellulitis)  Heart failure  Hyperglycemic hyperosmolar syndrome  Hypotension  Hypoglycemia  Malignant hypertension  Polyneuropathy |

^Excluded falls, accidents, lacerations or pain due to cancer or tumors

^#^Not related to myocardial infarction
